# Supplementary material for: Soft money, hard power: Mapping the material contingencies of change in global health academic structures
Source: PLOS Glob Public Health. 2025 May 28;5(5):e0004622. doi: 10.1371/journal.pgph.0004622 (PMC12118894; doi:10.1371/journal.pgph.0004622)
Supplement: S1 Text — (DOCX) [file pgph.0004622.s001.docx]

**Interview guide for [redacted] Faculty**

The anthropological model of semi-structured interviewing that we will follow lists topics in the interview guide is covered in a conversational manner, without specifying the order of particular questions or probes. This requires the researcher to introduce topics from the list in whatever order makes sense based on the flow of the conversation—this will vary somewhat from interview to interview. This requires active listening and consideration of what the respondent has already told the interviewer.

Tell me about your path to becoming a member of the [redacted] faculty. Where and how were you trained? What led to your specific research interests in global health?

What do you see as the main importance of the [redacted]? Who does it benefit the most? In what ways?

What do you see as the main purpose of your research and placement in this institution?

What do you consider are [redacted]’s obligations to the communities around the world they intervene in? What do you feel is the mission of this department?

What do you consider your research obligations to the communities you work in? How do you fulfill these obligations? Do you feel there are any obligations you are not able to fulfill, and if so, why?

What impact do you think your work has on the communities or spaces you work in?

Do you feel as though this impact is enough?

How much agency do you think you have to change the discipline?

Tell me a story about a time that you tried to create change, either inside the department or [redacted] broadly to change the structural conditions of those you work with abroad.

Do you feel like you are able to do the research and work you want to do? If not, what is the largest limiting factor to having impacts in the ways you want?

What are the biggest flaws to the [redacted] Department? Does your work inside and outside of research contribute to these flaws? Do you feel as though you work to address these flaws?

What are the biggest flaws to global health as a field of study and a practice aimed at improving the health of people around the world? Does your work contribute to these flaws? Does it seek to address these flaws?

Where are the key places change can be made inside the discipline of global health? What is currently being done that is not working?

Do you feel as though the critiques to global health from the outside and other disciplines are fair? Why or why not?

When did you first learn about “decolonialism”? What about “decolonialism” in global health?

Define “decolonial global health” for me.

When did you first learn about “decolonization”?

What were your first feelings when you heard about “decolonization” in Global Health?

Do you keep up with the decolonization literature? If so, how much, if not, why?

Do you feel as though your work and research relate or add to this mission? How or how not?

Are there risks to talking about this movement and ideology with other faculty? Do you feel as though you can only talk about it in certain spaces or with certain colleagues?

How do you feel about your work and your job in relation to this ideology and movement?

How do you find the funding structures at [redacted] and Global health more broadly?

If global health requires changing global structures and systems to fulfill its mission, how does your research or other work inside of the department work to create structural change?

Do you feel as though your positionality in the [redacted] faculty ranking system influences your ability to participate in this social movement in the ways that you want to? How so or how not?

How have you seen global health change over the time you have been a student and a scholar?

To you, what must the future of global health look like in order to achieve its mission?

What are you doing, if anything at all, to make this vision happen?

Do you feel as though global health is shifting as a discipline?

Do other disciplines inform your work and thinking? Do you engage with critical medical anthropology at all?
