# Supplementary material for: Soft money, hard power: Mapping the material contingencies of change in global health academic structures
Source: PLOS Glob Public Health. 2025 May 28;5(5):e0004622. doi: 10.1371/journal.pgph.0004622 (PMC12118894; doi:10.1371/journal.pgph.0004622)
Supplement: Attachment — Submitted filename: Review PGPH.docx [file pgph.0004622.s002.docx]

*Soft Money, Hard Power: Mapping the Material Contingencies of Change in Global*

*Health Academic Structures*

Thank you for the opportunity to review this important article, where Kurgman and Bayingana present an intriguing paradox - while academics situated in elite Global North institutions agree that funding systems should be fundamentally rearranged to provide direct funding to institutions in the Global South, this shift threatens their livelihoods and they fear the system cannot be changed. This paradox makes us rethink what it really means to “decolonize” Global Health, and what that might entail in practice.

Overall:

1. Make sure you are consistent about capitalising/not capitalising Northern and Southern
2. There are several run-on sentences in this paper - to increase readability for non-expert audiences, I would suggest making some of them shorter. E.g. see page 2 paragraph 3, sentence starting with: “While funding has begun to shift…”

Abstract:

1. I think it’s important to note somewhere in the introduction that this is a case study from a single institution (albeit major institution)
2. Suggested simplification of wording in the last sentence: instead of “*As thinking and action on the part of elite, northern actors that embraces the possibilities that emerge in loss of resources and helps facilitate this transfer that materially and symbolically disadvantages them”,* perhaps use some wording from your discussion: *“The disconnect between the ideology of these faculty—nearly all agreed that power and funding streams should shift to the Global South—and the ways that faculty articulated how they could not achieve this in practice because of the structures they were embedded within,…”*

Introduction:

1. Page 2 paragraph 4 - please define “studying up”

Methods:

2. Page 4 paragraph 2 - please define “hard money position”

Results:

3. Page 4 paragraph 3 - please change “*powerlessness”* to *powerless*
4. Page 4 paragraph 3 - “*the fact that they were materially dependent on them for their livelihoods.” - who is “they” and who is “them”?*
5. Page 5 paragraph 3 - by indirects do you mean indirect costs?
6. Page 6 paragraph 1 - remove *BSPH* (I presume the university isn’t supposed to be named)
7. Page 7 paragraph 5 - is there a reason the quote in this paragraph isn’t separated and italicised like the other quotes are?
8. Page 8 paragraph 4 - remove *BSPH*
9. Page 9 paragraph 1 - please put the full form of “IH” in *IH Department*
10. Page 10 paragraph 2 - remove *BSPH*
11. Page 10 paragraph 4 - right after the sentence where you say “*faculty expressed fear about a world where funding dramatically shifted*” - I suggest adding a line to express what that shift means. Is it that funding is routed entirely to institutions in Global South, at the expense of limiting IDC for the Global North? Or is it that both parties “get their 63%”?

Discussion:

1. To contextualise this research study in light of recent political events in the US - you can perhaps note somewhere in the discussion that budget cuts to research funding could exacerbate issues that have been highlighted in your study
2. Page 12 paragraph 3 - I’m not sure if you’ve used “GN” for Global North before - please replace with Global North, or explain the abbreviation somewhere above
3. I suggest adding a line to critique the potential limitations of your approach - this is a single centre study with a predominantly white American sample being interviewed, which could skew the results. However, this provides a fantastic starting point - it would be really interesting to see how the themes that emerged from your study compare to those in other institutions.

Conclusion:

1. Page 17 paragraph 1 - please put the full form of DEI
