# Supplementary material for: Soft money, hard power: Mapping the material contingencies of change in global health academic structures
Source: PLOS Glob Public Health. 2025 May 28;5(5):e0004622. doi: 10.1371/journal.pgph.0004622 (PMC12118894; doi:10.1371/journal.pgph.0004622)
Supplement: Attachment — Submitted filename: Response to Reviewers PLOS.docx [file pgph.0004622.s003.docx]

To Reviewers and Editors,

Thank you all for such productive and generative comments and edits to our manuscript. We found it to be an exciting and fruitful exercise going back through our draft and making changes. Our most significant alterations to the manuscript have been in writing style, scope, and clarification. We have taken significant steps to reduce length of sentences and clarify key points in concise terms as reviewers suggested. We have also clarified the limits of studying these dynamics at a single institution, narrowed what is able to be generalized from this data, and provided more significant citations of the existing literature on funding changes in the field. We believe that this article now more precisely builds from the data to produce the insights we are arguing for, as well as providing more clarification to what is being argued. Specific responses to each reviewer comments are given in red font below.

Reviewer #1

Overall:

Make sure you are consistent about capitalising/not capitalising Northern and Southern

This has been dutifully noted and standardized throughout the paper. Thank you.

There are several run-on sentences in this paper - to increase readability for non-expert audiences, I would suggest making some of them shorter. E.g. see page 2 paragraph 3, sentence starting with: “While funding has begun to shift…”

Run on sentences have been broken up and rewritten, including the one mentioned in this comment.

Abstract:

1. I think it’s important to note somewhere in the introduction that this is a case study from a single institution (albeit major institution)

This suggestion has been noted and the abstract now notes the study is at a single major institution.

2. Suggested simplification of wording in the last sentence: instead of “*As thinking and action on the part of elite, northern actors that embraces the possibilities that emerge in loss of resources and helps facilitate this transfer that materially and symbolically disadvantages them”,* perhaps use some wording from your discussion: *“The disconnect between the ideology of these faculty—nearly all agreed that power and funding streams should shift to the Global South—and the ways that faculty articulated how they could not achieve this in practice because of the structures they were embedded within,…”*

These sentences have been clarified and each has been shortened

Introduction:

1. Page 2 paragraph 4 - please define “studying up”

“Studying up” has been more defined here.

Methods:

2. Page 4 paragraph 2 - please define “hard money position”

“Hard money” has been defined here.

Results:

3. Page 4 paragraph 3 - please change “*powerlessness”* to *powerless*

This change has been made.

4. Page 4 paragraph 3 - “*the fact that they were materially dependent on them for their livelihoods.” - who is “they” and who is “them”?*

This clause has been clarified.

5. Page 5 paragraph 3 - by indirects do you mean indirect costs?

This has been clarified.

6. Page 6 paragraph 1 - remove *BSPH* (I presume the university isn’t supposed to be named)

This has been removed.

7. Page 7 paragraph 5 - is there a reason the quote in this paragraph isn’t separated and italicised like the other quotes are?

This has been standardized.

8. Page 8 paragraph 4 - remove *BSPH*

This has been removed.

9. Page 9 paragraph 1 - please put the full form of “IH” in *IH Department*

This has been removed.

10. Page 10 paragraph 2 - remove *BSPH*

This has been removed.

11. Page 10 paragraph 4 - right after the sentence where you say “*faculty expressed fear about a world where funding dramatically shifted*” - I suggest adding a line to express what that shift means. Is it that funding is routed entirely to institutions in Global South, at the expense of limiting IDC for the Global North? Or is it that both parties “get their 63%”?

This sentence has been clarified.

Discussion:

1. To contextualise this research study in light of recent political events in the US - you can perhaps note somewhere in the discussion that budget cuts to research funding could exacerbate issues that have been highlighted in your study

We have noted the potential shifts created by the Trump presidency in the conclusion to contextualize this work.

2. Page 12 paragraph 3 - I’m not sure if you’ve used “GN” for Global North before - please replace with Global North, or explain the abbreviation somewhere above

This has been standardized.

3. I suggest adding a line to critique the potential limitations of your approach - this is a single centre study with a predominantly white American sample being interviewed, which could skew the results. However, this provides a fantastic starting point - it would be really interesting to see how the themes that emerged from your study compare to those in other institutions.

This line of critique has been added to the conclusion, and we make clear how this article is supposed to be a basis for others to build from.

Conclusion:

1. Page 17 paragraph 1 - please put the full form of DEI

This change has been made.

Reviewer #2

1. I do not agree with the underlined words in the following statement, “how Global North institutions still largely dominate the field through money, how they have compelling financial interests to resist current calls for change, and how flows of funding influence how change is being conceptualized and what it can do remains unexamined.”

The influence of funding flows on change (or lack thereof) in colonialist power structures is, in fact, an active area of scholarship and critique in global health. Structural issues related to funding flows and incentives that reinforce Global North predominance have been examined by several different authors. I’d encourage these authors to read the papers below (and review the reference lists) and cite some or all of these references in your own paper at the appropriate points:

- Haberer J and Boum Y. Behind-the-Scenes Investment for Equity in Global Health Research. New England Journal of Medicine 2023;388:387-390.
- Oti SO (2024) Towards authentic institutional allyship by global health funders. PLOS Glob Public Health 4(3): e0003024. https://doi.org/10.1371/journal.pgph.0003024
- Cakouros BE, Gum J, Levine DL, Lewis J, Wright AH, Dahn B, Talbert-Slagle K. Exploring equity in global health collaborations: a qualitative study of donor and recipient power dynamics in Liberia. BMJ Glob Health. 2024 Mar 13;9(3):e014399. doi: 10.1136/bmjgh-2023-014399.

Thank you for sharing this literature and diving more into this area of literature. However, we want to note that almost everything argued for here has been substantially elucidated and argued for by anthropologists studying Global Health for a decade in the literature that we already cite, and our goal here is to transgress the “allyship” discourses pushed by each of these articles. We have significantly revised the introduction along the lines suggested, though.

2. I note several places where copy-editing would help improve the readability of this manuscript. Below are some examples (note that I did some copy-editing in CAPS, but other changes were not retained when I submitted this review through the PLoS GPH website):
- In the first sentence of the Abstract: “In the proliferating conversations about decolonizing Global Health, a basic assumption has been THAT Global South actors WOULD/WILL BE the primary conceptualizers, leaders, and undertakers of change with Global North counterparts thought of as allies or accomplices.”
- Second sentence of the abstract: “…this article argues that examining resource structures and material flows complicates assumptions about whose responsibility it is to “decolonize” what and how different actors should go about different actions considered to be decolonial.
- From the introduction: “With making power itself an object of study, this anthropological approach that investigates the cultures and financial structures of those in power, with the goal of illuminating how these structures are shaped, enabling better understanding their makeup and influence [11].
- From the results: “…most also saw themselves as powerlessness to make these changes happen given their size, historical embedding…”

I’m not going to mark all the copy-editing that is needed in this manuscript, but I encourage the authors to give the manuscript another thorough revision before resubmitting to improve its readability and clarity. (Might be worth paying a copy-editor to do this for you, perhaps a student who is a strong writer but not an expert in this field, who can help improve clarity for lay readers.)

We have thoroughly proofread the second draft of this manuscript and have had multiple sets of eyes review the manuscript to make sure this does not happen again.

3. Please make sure you are correctly using the words “Affects” (a verb) and “Effects” (a noun).

We note this suggestion. Our original use of affect following the use of the word following affect theory in social science and the humanities, which uses affect as a noun where effect should be used. Given that we don’t have room to explain this, we have eliminated this usage.

4. I’m not convinced that “Global Health” needs to be capitalized throughout this manuscript. To me, this is distracting, but I will accept it if the authors feel strongly.
The authors do feel strongly that the field that calls itself “Global Health” is not the same as the idea of “global health,” and conflation of the two is one of the reasons why change does not happen in the field.

6. Is “Marxist analysis” a specific anthropological method or framework? It is not cited, nor is it clear how/why this particular type of analysis is relevant or the best choice for this paper. Fine with me if you omit it in the next version, but if you choose to retain it, please cite/explain why.
We do not feel as though we have sufficient space to explain what we mean by Marxist analysis and what this entails so we have eliminated reference to it.

7. You refer to “BSPH” as the school or “the IH Department” where the interviews were conducted, but in other parts of this manuscript, you seem to be taking pains not to identify the “major Global North school of public health.” In one of the quotes, the respondent says “we’re Johns Hopkins.” All such identifying information should be removed to protect the anonymity of your respondents.
All references to the institution mistakenly included from a previous draft have been eliminated.

8. You refer to “the IDC system” without explaining what that is; please do not assume that your readers will understand this niche terminology. Also, please cite the Haberer and Boum piece when referencing inequities in indirect rates!

We are a bit confused about this comment. What the IDC system is has been clearly explained *through* our interlocutors’ words within the results section—remaining true to both the objective outline of what the system is and their experience of it. Further, while we have added the Haberer and Boum piece in throughout the article, we do not see how it is relevant in every instance of “referencing inequities in indirect rates” given that this article is a short think piece that does not build on the original research on indirect cost inequalities throughout the 2010s by social scientists cited elsewhere in this article. Nevertheless, we have taken this suggestion and have cited Harberer and Boum in the discussion and have further clarified what the IDC system is.

9. In your Discussion, you state: “By outlining how Global Health is a business where resources flow in particular ways in which more benefits, protections, and influence are granted to those higher up on the hierarchy, we have sought to open new conversations about social change in Global Health, what it must do, and who is responsible for it.” But this is a significant overstatement of what you have reported in this manuscript, and it undercuts the value of your work. You have not described the entire field of Global Health as a business, nor have you described the hierarchy of the entire field. Doing so would take many, many papers doing what you have done here as well as many other types of analyses. What you have done is more narrow but very important: you have provided primary data from Global North actors at a U.S. public health institution that captures their firsthand experience in a soft money funding structure that perpetuates power disparities and structural inequities. You don’t know, from your data, whether this description captures a broader phenomenon, but you can call for more research like your paper to explore that question. I urge you to rewrite the first paragraph of your discussion to summarize your findings without making such a grandiose claim.
While many have successfully established how Global Health is a business without many many papers (see Erikson 2015, the works of Johanna Crane, Anne Emmanel Birn, and Katerini Storeng, for example), this comment is correct and we have significantly reconfigured the first paragraph to reflect a more narrow frame.

10. You repeatedly refer to the Global North institution as “elite” or “most elite,” which I find off-putting. What do you mean by that? And why does it matter? Is the “elite” nature of the institution relevant to your findings? I find myself wanting you simply to report that you conducted this analysis at a public health school in the U.S. , and that, from your own positionality, you suspect that the phenomena you have identified may reflect common issues at many public health schools in the Global North (and you actually specifically mean public health schools in the U.S. – from my own research, I know that at least some Canadian and European institutions handle indirect costs and funding structures differently from U.S. institutions; you want to be careful to provide an appropriately limited description of what you claim to be studying—and to have learned—here).

We are not sure what to do with this comment. It seems self-evident to us in this milieu of knowledge about Global Health that institutions and people located in imperial cores—whether that be the United States, Canada, or Europe—are elites vis-à-vis Southern counterparts, and that within this cadre of elite institutions there are others who have more prestige and capital than others. We are not sure what the productivity of eliminating this qualifier of the kinds of institutions we are talking about would be, other than to acknowledge the fact that many public health institutions across the global South too have soft money structuring and this type of financial structuring is a system that was developed at a particular institution in the United States and has been spread around the world as the standard of what public health institutions should be. To be clear, we are not fundamentally talking about IDC systems, of which there are many different kinds and which rates vary by institution. We are talking about a financial system in which researchers must win grants to fund themselves and their institutions in which IDCs are only one part of the game. But given this, we have made clear that this is a study about the financial system at *one* institution in the United States, elucidating an underlying system that it is a chief representative of.

11. Again in the discussion, you overstate what you have demonstrated in your results, writing: “Here, we show how Global North actors are forced to reproduce colonial inequalities of their work when this already politically charged money is acquired in “soft money” funding schemes. As shown in the data, the survival of the individual researcher and the university are intertwined in the “soft-money” business model.” You did not conduct a study that explores enough Global North institutions (which includes many countries beyond the U.S.) to be able to make this claim. Many Canadian universities offer hard-money positions for their public health faculty. I think the same may be true of European institutions. It may also be the case that some U.S. universities offer hard-money positions for public health faculty; you do not know. Make sure that you describe your findings accurately and appropriately. Overstating your results discredits the validity of the entire study. You need to change this throughout the discussion.
We have significantly changed the discussion section to reflect this comment.

12. Your discussion section is undercited. Several of the dynamics and power differentials you mention have been discussed and called out openly in other publications – important to include those and honor the work of others who are doing this anti-colonial work. Please revise with a more thorough effort to cite the work of others who have called attention to these problems (see the papers I cited above as a good starting point for familiarizing yourselves with this literature).
We have significantly increased the number of citations in the discussion section from the literatures we see fit. As this is a qualitative study, our goal here is primarily to let the concerns and dynamics of our interlocutors speak.

13. The last paragraph of the section entitled “The Materiality of Power” reads like an opinion piece. It is not very grounded in the results you present earlier in the paper. Your tendency to overstate the breadth and reproducibility of your findings is not doing good service to the important work of this paper. Please revise to focus your discussion more on your own results and on how those results add to our understanding of these issues in global health.
This paragraph has been changed and now reads: “In sum, the structuring of this institution based on soft money is designed in a way that maintains inequities. While this is one, albeit centripetal, example of an institutional financial system, the soft money system outlined here is a baseline structure that has been replicated across the world In this financial structure as elucidated through this case, gaining vast grant money and prestige, the financial integrity of elite Global North institutions depends on this system in which their researchers must secure a disproportionate amount of the few grants that are available at the expense of their Global South partners.”

14. The following excerpt from the Discussion feels very important to me, but it is difficult to understand: “…the end of the need for the field as it exists as infrastructures are developed complete autonomy over health-creating research and interventions is gained in formerly colonized regions.” Please reword for clarity.

This has been reworded for clarity.

16. This is opinion only, for the authors’ consideration: I think the section entitled “The Double Bind of Change in the Global North” is very well-written and powerful. I’d suggest moving it up in the discussion and perhaps cutting some of the rest of the discussion section to emphasize the points made in this section. It feels strongly grounded in your own primary data and highly relevant to the takeaway points that you intend to make in this manuscript.
Thank you, this suggestion has been taken and the materiality of power section has been shortened.

17. The section “Rethinking Responsibility through Ruinous Solidarity” is interesting…it is written differently than the rest of the discussion, with many more direct quotes. I think the “ruinous solidarity” concept is worth raising, although I would prefer that you reach it more directly, with less lead-up and fewer direct quotes of other authors. I think, however, that my critique has to do with my training; I am not an anthropologist and prefer more direct writing. So, I give you this critique to consider: I’d like for this section to be pithier, which I think would make the contribution of the “ruinous solidarity” concept easier for your readers to grasp and therefore more powerful. But I defer to the authors on this point.

We have noted the reviewers point here and reach this analytic in quicker way. However, in line with the reviewers suggestion to cite more to “honor the work of others who are doing this anti-colonial work”, we keep many quotes of scholars whose words we directly follow (and believe are doing much more direct anti-colonial work than what is currently going on in Global Health). We hope this section is now clearer.

18. Having reached the end of your paper, I do think this is an important contribution to the literature and worth publication after revising, but your analysis is not about the Global North. It is specific to the U.S. and to a single U.S. university. Every funder you mention is a U.S. funder (NIH, CDC, USAID, Gates), and the issues you flag are unique to U.S. soft-money institutions and the way that those U.S. institutions manage/negotiate indirect cost coverage with their cognizant federal funding agency. The dynamics that your respondents describe are not universal to wealthy Global North institutions, and you need to be careful not to say so. Please revise the paper to indicate that your analysis focuses specifically on a U.S. university.

We fundamentally disagree with this argument and, while making clear how this is a particular study in the United States, do not take up this suggestion. The financial system where health researchers must get grants to fund themselves and their institutions is a phenomenon now seen around the world. What we have done is shown the workings and *affects* (that is, the feelings and thinking it creates for the people it subjects) of the *original* US version of this system—the one that has been transported across the world originating from the very institution we study (see Fee’s 2016 book). We do not claim that the dynamics we unravel here are universal to every Global Health researcher everywhere, nor that every soft money system will be the same at every institution will be the same. We have elucidated what this *structure* institutionalized idea of soft money—one that extends far beyond the walls of the institution that we study—creates particular kinds of subjects, feelings, actions and ways of thinking about changing the field. This is not about funders or even the institution itself. It is about soft money as the normative financial structure of the field. However, we do make clear that this is reflective of the US system and will be much different in other Global North nations, although, as reviewer #3 notes in contrast to this review, this soft money system has increased in Europe due to neoliberal austerity.

Reviewer #3


2) I have some minor comments: whenever abbreviations are introduced please spell them out in full such as IDC, CDC, NIH etc. Also the IDC system needs more clarification - what is the 63% and why is it at this rate? Are the Universities in need of this money from grants because of the systematic reduction of grants from the government [as has happened with public Universities in Europe?] For readers not familiar with the system this description would be helpful.

We have clarified what IDC grants are, but do not feel as though we have sufficient space to explain the history of this system (which is fascinating and needs an article in its own right).

3) While everywhere else in the manuscript the authors mention elite University in GN, there's one place where the institution is mentioned by one of the respondents - might be good to redact it unless they want to keep it.

This was an accident and has been eliminated.

4)There's one place where the sentence needs clarification - "Given the history of Global Health, this is a logical and fruitful departure point for change. In a field grounded in coloniality [4] and productive of vast epistemic injustice [5], those who have been cast to the margins of the field, are closest to manifestations of health inequity, and have the most intimate relation with other epistemologies and ways of being should be privileged actors in efforts changing unequal political dynamics. However, this earnest vision of responsibility is one that departs from particular ideas of power. Reflecting broader trends in the field, critiques of ideological power—who shapes thinking and who’s knowledge is taken up—and symbolic power—tacit modes of Western domination reproduced through habits of doing Global Health
work—disproportionately shape how this responsibility is conceptualized. That is, departing from the perspectives, the field can be changed through shifting thinking, desires, and actions, and power can be democratized between North and South when ideologies, knowledges, and
habits change. As anthropologists and other critical scholars have revealed". Here what does the particular ideas of power mean? Is it the previous sentence or the sentence that follows it? Because both are critiques. I found this bit a little confusing.

This paragraph in the introduction has been rewritten and changed.

5) While this is not part of your argument, it might be good to mention that decolonial approaches often are unable to solve the problem of the north within the south. Even when money trickles from GN institutions, elite actors in GS institutions [and elite institutions] end up reaping the benefits.

Given that reviewer #2 is pushing us to keep attention tighter to what our data reveals, we cannot extend our thinking to this very correct and needed realm. Both author’s work are working on this problem in different ways, though.
