# Supplementary material for: Soft money, hard power: Mapping the material contingencies of change in global health academic structures
Source: PLOS Glob Public Health. 2025 May 28;5(5):e0004622. doi: 10.1371/journal.pgph.0004622 (PMC12118894; doi:10.1371/journal.pgph.0004622)
Supplement: Attachment — Submitted filename: PLOS_response_3.docx [file pgph.0004622.s005.docx]

PLOS GPH Response to Reviewers

To the reviewers and editors,

Thank you for a favorable review. We have edited to make sure that all abbreviations are spelled out on their first instance.

We have also edited the conclusion to include how this research speaks to the present moment in the wake of the Trump administration changes.

Thank you all,

DK & AB
