# Supplementary material for: Soft money, hard power: Mapping the material contingencies of change in global health academic structures
Source: PLOS Glob Public Health. 2025 May 28;5(5):e0004622. doi: 10.1371/journal.pgph.0004622 (PMC12118894; doi:10.1371/journal.pgph.0004622)
Supplement: Attachment — Submitted filename: PLOS Response_to_reviewrs_3.docx [file pgph.0004622.s007.docx]

To the Editors of PLOS Global Public Health,

Thank you for these hopefully final comments. Please see our response to each point.

1) We have cleaned up our in-text citations and bibliography section. We found that the number of in text citations was more than the number of actual citations. Thus, we did not eliminate any citations from the piece and, rather, just renumbered the existing citations.
2) We are greatly confused about this request, as article 3 of PLOS GPH’s publication criteria seems to be about qualitative RCTs and statistical explorations. This is a qualitative study, and unlike quantitative ones, replicability is not a principle that demonstrates the rigorousness of the study. Qualitative research by design cannot be fully replicable, as the social contexts and the people a study engages with are fluid across contexts. It is highly dependent upon the interpretation of the research teams and how they approach the data as well. Unlike quantitative research that seeks to produce results through methodology that can be reproduced, no matter how many times this study is done by different researchers, some kind of different results will be produced. By the requirements of our IRB, we cannot share the interview guide, as it would reveal the institution this study was undertaken at. Further, even if our guide was shared, its ability to be used in different places or used again at this institution does not prove the rigorousness of the study, and demanding this of qualitative research defeats the purpose of it. The goal of qualitative research is to draw out salient features of social life in a place and group of people to be further interrogated and explored—not to produce an objective fact that can be confirmed by others doing the same study. If there are specific things that need to be added to the methods section, please let us know. But given that reviewers, who we assume were fellow qualitative researchers, did not have suggestions beyond what we already added to it, we do not feel this needs changing at the moment.
3) In contrast to replicability, generalizability is a virtue of good qualitative research, and we have dutifully considered your push to recognize this more. Our conclusion now features our limitations and calls for more research in the areas where our study is limited.
